# Short hairpin-looped oligodeoxynucleotides reduce hepatitis C virus replication

**DOI:** 10.1186/1743-422X-9-134

**Published:** 2012-07-23

**Authors:** Felix Broecker, Karin Moelling

**Affiliations:** 1 University of Zurich, Gloriastrasse 30/32, CH-8006 Zurich, Switzerland; 2 Max Planck Institute for Molecular Genetics, Ihnestrasse 63-73, 14195, Berlin, Germany; 3 Max Planck Institute of Colloids and Interfaces, Department Biomolecular Systems c/o Free University of Berlin, Arnimallee 22, D-14195, Berlin, Germany; 4 Heinrich Pette Institute, Department of Virology, Martini Street 52, D-20251, Hamburg, Germany

**Keywords:** HCV, Hepatitis, Replicon, Oligodeoxynucleotide, ODN, Broad inhibitor, Antiviral therapy

## Abstract

**Background:**

Persistent infection with hepatitis C virus (HCV) is a leading cause of chronic hepatitis, liver cirrhosis, and hepatocellular carcinoma. Standard therapy consists of a combination of interferon-alpha and ribavirin, but many patients respond poorly, especially those infected with HCV genotypes 1 and 4. Furthermore, standard therapy is associated with severe side-effects. Thus, alternative therapeutic approaches against HCV are needed.

**Findings:**

Here, we studied the effect of a new class of antiviral agents against HCV, short, partially double-stranded oligodeoxynucleotides (ODNs), on viral replication. We targeted the 5’ nontranslated region (5’ NTR) of the HCV genome that has previously been shown as effective target for small interfering RNAs (siRNAs) *in vitro*. One of the investigated ODNs, ODN 320, significantly and efficiently reduced replication of HCV replicons in a sequence-, time- and dose-dependent manner. ODN 320 targets a genomic region highly conserved among different HCV genotypes and might thus be able to inhibit a broad range of genotypes and subtypes.

**Conclusions:**

ODNs provide an additional approach for inhibition of HCV, might be superior to siRNAs in terms of stability and cellular delivery, and suitable against HCV resistant to standard therapy. This study underlines the potential of partially double-stranded ODNs as antiviral agents.

## Findings

More than 170 million people worldwide are persistently infected with hepatitis C virus (HCV). HCV infection is an emerging threat and a major risk factor in the development of chronic liver disease, cirrhosis and hepatocellular carcinoma (HCC) [1].

HCV is an enveloped virus with a single-stranded ~9.6 kb RNA genome of positive polarity, classified as member of the *Flaviviridae* family [2,3]. The 5’ nontranslated region (NTR) of the genome contains an internal ribosome entry site (IRES) that directs translation of a single long polyprotein, which is post-translationally cleaved into 10 viral proteins (Core, E1, E2, p7, NS2, NS3, NS4A, NS4B, NS5A, and NS5B). The HCV genome exhibits a high degree of genetic diversity. Based on nucleotide sequences, HCV is grouped into at least six genotypes that differ from each other by 31 to 33% [4]. In the United States and Europe, genotype 1 is the most prevalent, followed by genotypes 2 and 3. Other genotypes are found mostly in specific geographical regions, Egypt (genotype 4), South Africa (genotype 5) and Southeast Asia (genotype 6). The extensive genetic diversity is due to the high error rate of the viral RNA polymerase of ~10^−4^ errors per nucleotide [5].

HCV therapy is currently limited to interferon-alpha (IFN-α) alone or combined with ribavirin, which is successful in less than 50% of patients infected with genotype 1 HCV and associated with severe side effects [6,7]. Poor response to standard therapy, especially of genotypes 1 and 4, poses a major challenge [7,8]. Thus, alternative therapeutic approaches for chronic HCV infection are needed.

We have recently explored a new class oligodeoxynucleotides, partially double-stranded hairpin loop-structured ODNs, as sequence-specific antiviral agents. ODNs consist of a 25-mer antisense strand fully complementary to the target RNA, a linker of four deoxythymidines, and a 25-mer second strand partially complementary to the antisense strand.

We have demonstrated efficacy of ODNs *in vitro* against HIV-1 [9-11], herpes simplex virus-1 (HSV-1) [12] and BK virus (unpublished data), and *in vivo* against HIV-1 [13], spleen focus forming virus (SFFV) [14] and influenza A virus [15]. We showed that ODNs can inhibit HIV-1 replication in humanized HIV-infected SCID mice, and were even able to prevent infection [13]. ODNs directed against telomerase could reduce tumor formation in mice [16], underlining their broad applicability. We here report that ODNs can also inhibit HCV replication in cell culture.

**Figure 1:**
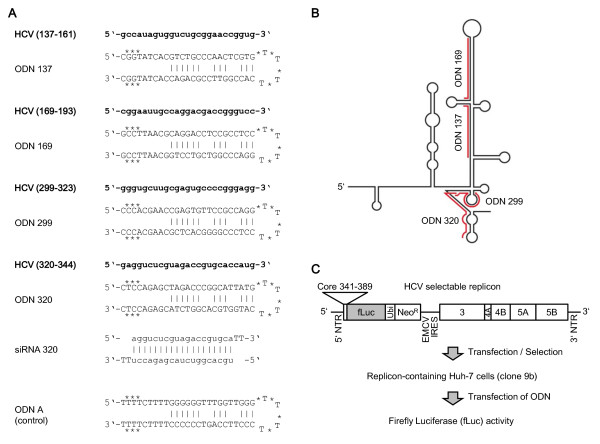
**(A) Sequences of the ODNs used in this study, (B) target regions on the HCV 5’ NTR, and (C) experimental strategy using the replicon system.** Huh-7 clone 9b cells stably expressing the HCV I_389_/NS3-3’/LucUbiNeo-ET replicon were kindly supplied to us. Stars in (**A**) mark phosphorothioate bonds. Figure (**C**) adapted from [21].

The sequences of the antisense strands of the ODNs used here were based on known small interfering RNAs (siRNAs) targeting the 5’ NTR of the viral genome, that have recently been shown to significantly inhibit HCV replication [17-19]. The ODN sequences are shown in Figure 1A, and their target regions on the HCV 5’ NTR, which forms extensive secondary structures, in Figure 1B. As negative control we used a structurally identical ODN without any sequence similarity to the HCV genome (ODN A in Figure 1A). ODNs were protected against nucleases by phosphorothioate modifications at both ends and at the deoxythymidine linker (Figure 1A, stars), as described [9,20]. ODNs were purchased from Integrated DNA Technologies.

To assess antiviral efficacy of anti-HCV ODNs, we used a well-established replicon system, I_389_/NS3-3’/LucUbiNeo-ET, stably expressed by human hepatoma Huh-7 clone 9b cells, as described [21] (Figure 1C). This replicon contains all non-structural HCV genes (3, 4A, 4B, 5A, 5B) necessary for viral replication, under control of the encephalomyocarditis virus (EMCV) IRES, as well as the Core protein, firefly Luciferase (fLuc) and neomycin phosphotransferase (Neo^R^) genes under control of the HCV 5’ NTR IRES, and the HCV 3’ NTR. Neo^R^ confers resistance to G418, which was used to select replicon-containing cells, and fLuc activity was used to quantitate HCV replication, as described [21] (see experimental strategy in Figure 1C). We used the Dual Luciferase Reporter Assay System (Promega) to determine fLuc activities, according to the manufacturer’s recommendations, in a Sirius luminometer (Berthold Detection Systems). Replicon-expressing Huh-7 clone 9b cells were maintained in Dulbecco’s Modified Eagle Medium (DMEM) (Invitrogen) supplemented with 10% fetal calf serum (Brunschwig), 100 U/ml penicillin and 100 μg/ml streptomycin (Invitrogen) and 0.5 mg/ml G418 (Life Technologies).

To assess their antiviral effect, we transfected replicon-expressing Huh-7 cells with ODNs in different concentrations. 24 h prior to transfection, freshly passaged cells were resuspended in antibiotics-free DMEM with 10% FCS and transferred into 12-well plates in a volume of 1 ml per well and grown to 70–90% confluency. We used Lullaby transfection reagent (OZ Biosciences) according to the manufacturer’s recommendations.

**Figure 2:**
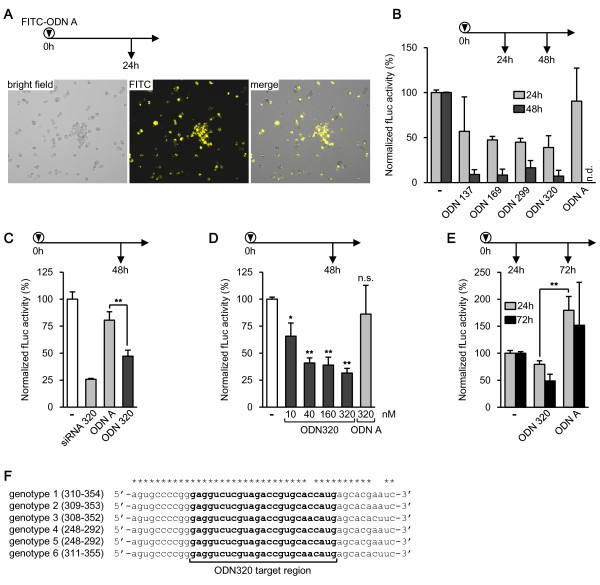
**Inhibition of HCV replication by ODNs assessed by replicon-expressing Huh-7 clone 9b cells.** In figures (**A**) to (**E**), 6 × 10^4^, 4 × 10^4^ or 2 × 10^4^ cells were seeded for 24 h, 48 h, or 72 h of incubation, respectively, as indicated above the graphs, 24 h prior to transfection as described in the text. ‘-‘denotes untransfected control cells otherwise treated identically. (**A**) Cells were transfected with FITC-labeled ODN A. After 24 h, cells were detached by trypsinization, paraformaldehyde-fixed and investigated with an inverted Leica SP5 fluorescence microscope. FITC was excited at 488 nm. (**B**) to (**E**), cells were transfected with 160 nM of the indicated ODNs, unless noted otherwise. Levels of significance were determined by two-sided Student’s t-Tests, *P≤0.05, **P≤0.01, against ODN A in (**C**) and (**E**), and against untransfected control cells in (**D**). Bars indicate mean values ± S.E.M of at least two independent experiments in duplicate. (**F**) Sequence comparison of the ODN 320 target region in the HCV 5’ NTR (bold) and flanking sequences, among genotypes 1 to 6. Accession numbers are [GenBank:AF009606], [GenBank:NC_009823], [GenBank:NC_009824], [GenBank:NC_009825], [GenBank:NC_009826], [GenBank:NC_009827] for genotypes 1 through 6. n.d., not determined; n.s., not significant (*t*-Test).

The transfection efficiency was assessed with a fluorescein isothiocyanate (FITC) labeled ODN A at a concentration of 160 nM. Transfection efficiency was greater than 90% (Figure 2A). We initially screened the four ODNs directed against the HCV genome (Figure 1A) for their efficacy to inhibit HCV replication, as assessed by fLuc reporter activity after cell lysis. All four ODNs at 160 nM inhibited HCV replication within 24 h and, more pronounced, within 48 h (Figure 2B). Since we noticed that cell growth was inhibited by the transfection procedure in this initial screen, we reduced the amount of Lullaby from 8 μl/ml to 4 μl/ml for the following experiments. When we used the lowered amount of Lullaby, neither cell morphology nor growth was affected, as assessed microscopically and by protein determination of the samples by Bradford assay (Bio-Rad GmbH). Protein contents of cell lysates of transfected vs. untransfected cells were comparable after 24, 48, and 72 h of incubation (data not shown). We concluded that there was little or no cytotoxicity of the transfection procedure using 4 μl/ml of Lullaby.

We chose ODN 320 for further experiments, since it showed the most efficacious inhibition of HCV replication in the initial screening (Figure 2B). fLuc activities were normalized to the protein contents of the respective lysates. ODN 320 at 160 nM reduced HCV replication to 47% after 48 h, with statistical significance (*P*≤0.01, Student’s *t*-Test) to control ODN A at the same concentration (Figure 2C). As positive control, we used an siRNA directed against the same target, siRNA 320 (purchased from Dharmacon) (Figure 1A), which also reduced HCV replication (Figure 2C). Next we assessed dose-dependency of ODN 320, with concentrations ranging from 10 to 320 nM. A dose-dependent inhibition of HCV replication was detected (Figure 2D). At 320 nM, ODN 320 reduced HCV replication to 32%, with statistical significance against untransfected cells (*P*≤0.01). ODN A at the same concentration did not inhibit HCV replication, demonstrating the sequence-specificity of the approach (Figure 2D). To assess short- and long-term effects of ODN 320, we transfected ODN 320 at 160 nM and determined fLuc activities after 24 and 72 h, respectively. ODN 320 inhibited HCV replication within 24 h, with statistical significance (*P*≤0.01 compared to ODN A) and, more pronounced, within 72 h (Figure 2E).

Due to the high genomic diversity of HCV, a conserved target sequence of the ODN is important to guarantee efficacy against different genotypes and subtypes. Thus we assessed the sequence diversity of the ODN 320 target region, nucleotides 320–344 on the viral genome, by comparing sequences of HCV genomes of strains 1 to 6. Remarkably, the target for ODN 320 is highly conserved among genotypes (Figure 2F). Only one C-to-A mutation was detected within this region in genotypes 3 and 6. Since one mismatch is likely tolerated, ODN 320 is an antiviral agent that is potentially efficacious against all genotypes of HCV. The replicon used in this study is based on the sequence of HCV genotype 1 subtype b [21]. Future investigations should aim to assess whether ODN 320 is indeed efficacious against other genotypes as well. Otherwise the ODN 320 can be modified to fully match the sequence.

HCV mainly replicates in the cytosol of hepatocytes, and liver-specific *in vivo* delivery of therapeutic nucleic acids is needed for their administration in conceivable therapeutic doses [22]. In recent years, promising advances in the liver-specific delivery of siRNAs have been made, using for instance modifications with lipophilic molecules such as cholesterol, bile-salt derivatives, fatty acids, or vitamin E, which promote binding to lipoproteins and internalization by lipoprotein receptors on liver cells (reviewed in [22]). These approaches might well be suitable for the delivery of ODNs as well, since they are biochemically and structurally highly related to siRNA duplexes [23].

Here we are demonstrating that a new class of oligodeoxynucleotides, partially double-stranded ODNs with one strand completely complementary to the target RNA, can reduce HCV replication *in vitro*. The results are consistent with previous studies reporting that ODNs have an antiviral effect against HIV-1, SFFV, influenza A virus, HSV-1 [9-15], and BK virus (unpublished data). The modes of action of ODNs in this study may include steric hindrance of ribosomes and creating a substrate for cellular RNases H, comparable to single-stranded antisense DNA molecules [24]. It was recently shown that the effect of ODNs is partially dependent on cellular RNases H [25]. Also, the mechanism of action is reminiscent of the siRNA machinery. The effect of siRNAs is mediated by Argonaute proteins, which have an RNase H-like structure [23]. However, in comparison with siRNAs, ODNs are superior in terms of stability, due to higher nuclease resistance [20,25], as well as cellular delivery (unpublished observation). ODNs are especially suitable to target drug-resistant virus variants, through selection of highly conserved target regions. For instance, it has been shown that an ODN directed against a conserved region of the HIV-1 genome efficiently abrogated infectivity even of drug-resistant HIV-1 isolates [11]. ODN 320 studied herein targets a highly conserved region in the otherwise highly variable HCV genome [4] (Figure 2F). Thus, ODN 320 may be suitable as antiviral agent against different strains of HCV, including those resistant to standard therapy, especially genotypes 1 and 4 [7,8]. Overall, this study underlines the potential of partially double-stranded ODNs as antiviral agents.

## Abbreviations

DMEM: Dulbecco’s Modified Eagle Medium; EMCV: encephalomyocarditis virus; FCS: fetal calf serum; FITC: fluorescein isothiocyanate; fLuc: firefly Luciferase; HCC: hepatocellular carcinoma; HCV: hepatitis C virus; HSV-1: herpes simplex virus-1; HIV-1: human immunodeficiency virus-1; IFN-α: interferon-alpha; IRES: internal ribosome entry site; NeoR, neomycin phosphotransferase; n.d.: not determined; n.s.: not significant; NTR: nontranslated region; ODN: oligodeoxynucleotide; RNase H: ribonuclease H; S.E.M: standard error of the mean; siRNA: small interfering RNA; SFFV: spleen focus forming virus.

## Competing interests

The authors declare no competing interests.

## Authors’ contributions

KM initiated this study. FB performed the experiments with assistance from KM. FB and KM prepared the manuscript. All authors read and approved the final version of the manuscript.

## Author details

^1^University of Zurich, Gloriastrasse 30/32, CH-8006 Zurich, Switzerland. ^2^Max Planck Institute for Molecular Genetics, Ihnestrasse 63-73, 14195 Berlin, Germany. ^3^Max Planck Institute of Colloids and Interfaces, Department Biomolecular Systems c/o Free University of Berlin, Arnimallee 22, D-14195 Berlin, Germany. ^4^Heinrich Pette Institute, Department of Virology, Martini Street 52, D-20251 Hamburg, Germany.
